# Ultra-high-dose-rate pulsed irradiation and the conditional FLASH effect: dosimetry, multiscale biology, and clinical translation

**DOI:** 10.3389/fonc.2026.1990706

**Published:** 2026-10-07

**Authors:** Jincheng Zhang, Hao Lu, Guofu Dong, Changzhen Wang

**Affiliations:** 1 Academy of Military Medical Sciences, Beijing, China; 2 School of Basic Medical Sciences, Anhui Medical University, Hefei, Anhui, China

**Keywords:** clinical translation, dosimetry, flash effect, normal-tissue sparing, pulsed irradiation, radiobiology, ultra-high dose rate

## Abstract

Ultra-high-dose-rate (UHDR) pulsed irradiation can deliver therapeutic doses within an extremely short time window, but UHDR is a physical delivery state, whereas the FLASH effect is a conditional comparative phenotype in which normal-tissue injury is reduced relative to a spatially dose-comparable conventional-dose-rate (CDR) irradiation comparator without loss of tumor control. This Review integrates evidence across four levels: physical exposure characterization, dosimetric measurement, biological comparison, and clinical translation. We examine the temporal structure and quality-assurance requirements of electron, very-high-energy electron, photon, proton, and heavy-ion UHDR platforms; synthesize positive, negative, and conditional findings across normal-tissue and tumor models; and integrate mechanistic studies spanning multiple time scales. Current evidence does not support 40 Gy/s, or any single average dose-rate metric, as either a sufficient or a necessary threshold for the FLASH effect. Effect magnitude and reproducibility are more likely determined jointly by dose per pulse, pulse structure, total delivery time, fractionation, irradiated volume, radiation quality, tissue-specific radiobiological characteristics, and biological state. Mechanistic data likewise favor multiscale coupling across time rather than a single explanatory pathway, although several causal links from early oxygen and radical dynamics to cellular and tissue outcomes still lack direct interventional validation. Human studies have established the feasibility of clinical UHDR delivery on selected electron and proton platforms and have provided preliminary safety data, but comparative clinical benefit over CDR irradiation has not yet been demonstrated. Further translation should therefore prioritize pulse-resolved traceable dosimetry, organ-specific normal-tissue dose-response relationships, late toxicity, comparable tumor control, and concurrent comparative clinical endpoints, with expansion guided by prespecified stage-gated translational criteria.

## 1 Introduction

Improving the therapeutic ratio remains a central objective of contemporary radiation oncology. Image guidance, intensity modulation, stereotactic techniques, and particle therapy have progressively improved spatial dose conformality and individualized treatment, yet the tension between the dose required for tumor control and the tolerance of organs at risk continues to constrain further widening of the therapeutic window (1). Ultra-high-dose-rate (UHDR) pulsed irradiation introduces dose rate, pulse structure, and total delivery time as additional optimization variables, extending radiotherapy from predominantly spatial conformality toward integrated dose-space-time control (2). In 2014, Favaudon et al. reported that UHDR electron irradiation reduced normal-lung injury without compromising tumor control in mouse lung and tumor models, catalyzing broad interest in the FLASH effect as a radiobiological phenomenon (3). Subsequent large-animal and companion-animal studies expanded both the species scale and irradiated-volume scale and also emphasized that late toxicity must be evaluated independently through long-term follow-up, particularly after high single doses and irradiation of large volumes (4, 5).

UHDR irradiation and the FLASH effect must be distinguished rigorously. The former describes a physical state of beam delivery; the latter is a comparative biological phenotype, observed relative to conventional-dose-rate (CDR) irradiation under spatially dose-comparable conditions, in which normal-tissue injury is reduced while tumor control is preserved (6). Throughout this Review, we use the term “conditional FLASH effect” to emphasize the dependence of the phenotype on irradiation parameters and biological context and to provide an analytical framework for organizing and interpreting the evidence. The frequently cited value of 40 Gy/s that emerged from early studies should be regarded as an operational reference rather than a sufficient or necessary biological threshold. Negative findings above this value and positive findings below it both indicate that a single average dose-rate metric cannot adequately represent the true exposure (7).

CDR effects primarily involve time-dependent biological processes such as damage repair and reoxygenation. Under UHDR conditions, however, extremely short delivery times and specific pulse structures may additionally introduce distinct radiation-chemical and oxygen-dynamic processes. Biological responses under UHDR irradiation therefore cannot be inferred by simple continuity from low-or CDR irradiation; they require direct experimental testing under clearly characterized and physically comparable exposure conditions (8).

As the evidence base has matured, the key question has shifted from whether a FLASH effect exists to which combinations of device, irradiation parameters, tissue, and endpoint can reproducibly generate relative normal-tissue sparing while maintaining tumor control in direct comparison (9). Addressing this question requires simultaneous consideration of physical exposure, dosimetric uncertainty, organ-specific biology, tumor control, and clinical-trial design (10). The accumulation of positive, negative, and conditional results further demonstrates that the FLASH phenotype is strongly dependent on experimental conditions and biological background. Recent reviews likewise indicate that irradiation parameters, *in vitro* and *in vivo* microenvironments, tissue oxygenation, and immune status may all modulate whether the phenotype occurs and how large it is (9, 10). Radiobiological parameters, including tissue- and tumor-specific fractionation sensitivity and α/β ratios, represent an additional component of this biological context and should be considered when interpreting differences across experimental systems (11, 12). Accordingly, defining the physical-biological parameter boundaries within which the FLASH effect appears, attenuates, or disappears is more informative than searching for a single dose-rate threshold that is universal across devices, radiation modalities, and organs. The clinically relevant question is whether those conditions yield a reproducible and meaningful gain in therapeutic ratio.

Against this background, this Review focuses on therapeutically relevant pulsed UHDR irradiation. We first define the temporal structure of UHDR delivery and the comparative conditions required to identify a FLASH phenotype. We then review the delivery characteristics and dosimetric foundations of electron, very-high-energy electron, photon, proton, and heavy-ion platforms and compare positive, negative, and conditional evidence across normal-tissue and tumor models. Mechanistic findings are subsequently integrated across time scales, including oxygen and radical dynamics, DNA damage and cell fate, redox and mitochondrial homeostasis, immunity, vascular responses, and tissue repair and remodeling. Finally, we use the available human evidence to define the current evidentiary limits of FLASH radiotherapy (FLASH-RT) and the validation steps required before broader clinical expansion.

## 2 Conceptual boundaries and evaluation criteria for UHDR irradiation and the FLASH effect

### 2.1 UHDR delivery parameters and criteria for identifying the FLASH effect

Average dose rate is commonly calculated by dividing total dose by a macroscopic irradiation time, but this metric compresses critical information about intra-pulse dose rate, dose per pulse (DPP), pulse number, and pulse interval. Two irradiation schemes can therefore have the same average dose rate yet create markedly different radiation-chemical environments because of differences in pulse width or beam-off intervals (13). In proton pencil-beam scanning, space and time are tightly coupled: each voxel may have a different start and end time of dose accumulation, scan sequence, energy-layer switching pattern, and repeated exposure, such that a single plan can correspond to multiple definitions of local dose rate (14). **Figure 1** summarizes these temporal descriptors and highlights the logical distinction between a UHDR physical exposure and a comparative FLASH phenotype.

**Figure 1:**
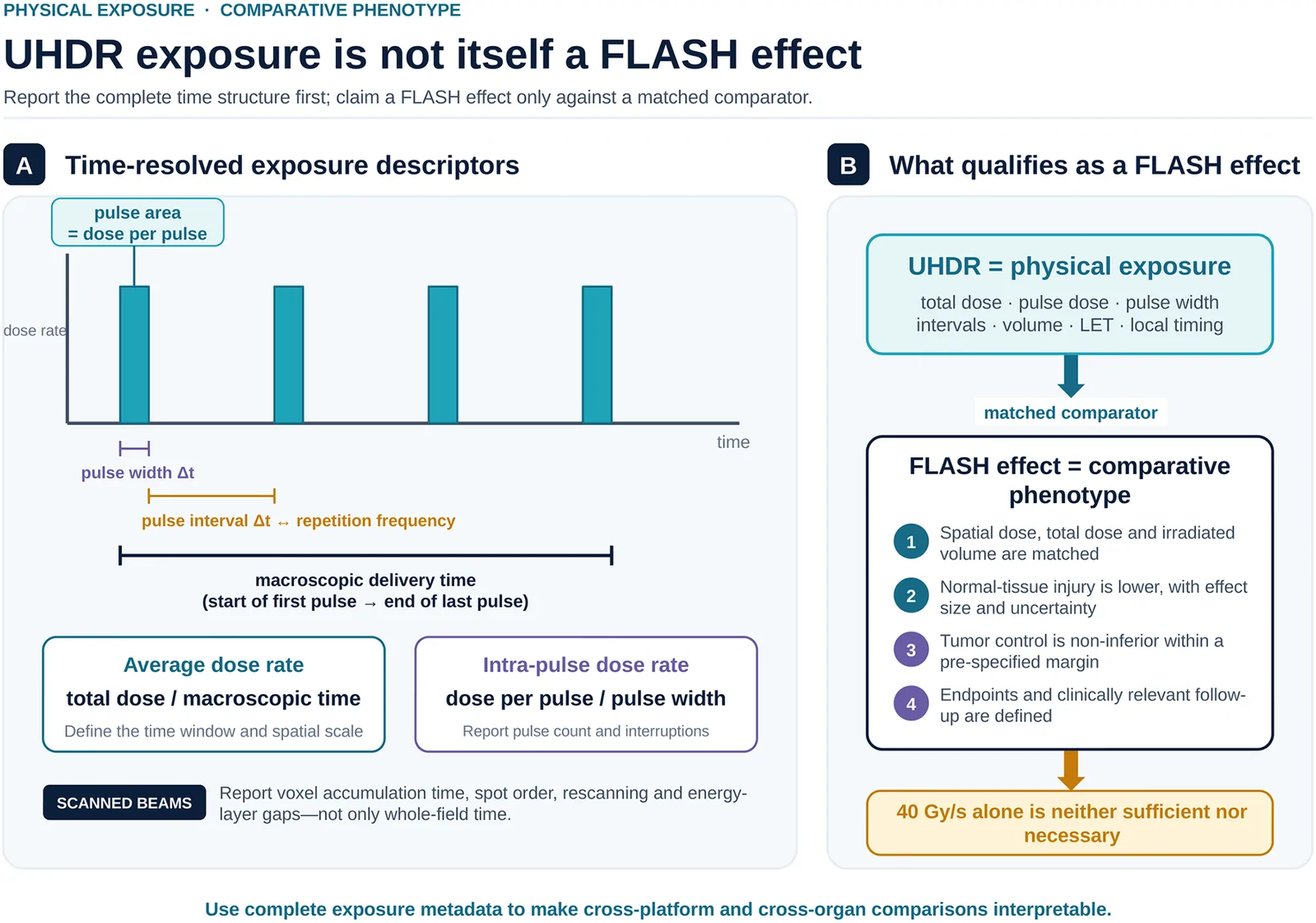
Temporal descriptors of UHDR physical exposure and the comparative boundary for identifying a FLASH effect. **(A)** Pulsed UHDR exposure should report at minimum DPP, pulse width, pulse interval/frequency, pulse number, total beam-on time, and macroscopic total delivery time; scanning beams should additionally document voxel dose-accumulation time, spot sequence, rescanning, and energy-layer switching. Average dose rate and intra-pulse dose rate are distinct descriptors. **(B)** UHDR itself is only a physical exposure. A comparative FLASH phenotype requiring explanation is present only when reduced normal-tissue injury is observed relative to a CDR comparator with comparable spatial dose, total dose, and irradiated volume, while tumor control remains comparable and clinically relevant endpoints/follow-up are defined. A value of 40 Gy/s alone is neither sufficient nor necessary. The term “non-inferior” for tumor control should be reserved for formal clinical designs with a prespecified statistical margin (6, 7, 10, 13, 17).

Time-resolved scintillator measurements further show that scan trajectories and local re-irradiation can generate complex dose-rate peaks and troughs, making a plan-level global average a poor representation of the transient process actually experienced by a tissue voxel (15). Machine log files can be used to reconstruct local dose and dose-rate distributions, but their reliability depends on timestamp precision, beam modeling, and independent measurement-based validation (16). Any reported dose-rate value should therefore specify the start and end points used in its calculation, the spatial scale, whether beam interruptions are included, and whether the metric is field-averaged, voxel-based, dose-weighted, or percentile-based.

### 2.2 Evidence types and effect evaluation

Once the physical delivery parameters have been defined, the questions that different study models can answer must also be distinguished. Prospective human studies primarily evaluate clinical feasibility, safety, and patient outcomes; large-animal, veterinary, and inter-institutional replication studies are particularly useful for testing scale-up and reproducibility; immunocompetent small-animal studies are commonly used to establish organ-specific dose-response relationships; and organoid, cellular, cell-free, and computational systems are most appropriate for generating and testing candidate mechanisms. This hierarchy is not intended as a formal grading of evidence. Rather, it prevents mechanistic associations, animal tissue sparing, and human clinical benefit from being treated as interchangeable forms of evidence.

Normal-tissue sparing should preferably be characterized using complete dose-response curves or dose ratios at equivalent levels of injury with corresponding confidence intervals, rather than inferred solely from statistical significance at a single dose point. Systematic evaluation of animal studies suggests that proton or carbon-ion UHDR irradiation may show trends toward tissue sparing, but between-study heterogeneity, small sample sizes, and publication bias limit the reliability of pooled conclusions (17). **Table 1** summarizes a minimum reporting set for UHDR/FLASH studies based on current reporting recommendations and consensus guidance, with the aim of improving completeness of physical exposure characterization and cross-study comparability (18).

**Table 1:** Minimum reporting information for reproducible UHDR/FLASH studies.

Dimension	Minimum items to report	Interpretation and considerations
Spatial dose and radiation quality	Total dose; dose per fraction; fractionation; depth-dose distribution; irradiated volume/field; energy spectrum; LET-related metrics	FLASH comparisons first require comparable spatial dose, total dose, and irradiated volume.
Temporal structure	Definition and time window for average dose rate; DPP; intra-pulse dose rate; pulse width; repetition frequency; pulse number; pulse interval; beam-on time; total delivery time	State whether pauses, scanning, and energy-layer switching are included. Average dose rate cannot substitute for DPP or intra-pulse dose rate.
Local exposure for scanned beams	Spot sequence; dwell time; voxel dose-accumulation start/end; rescanning; energy-layer switching; local overlapping exposure	For PBS and other scanning techniques, report local voxel-level temporal structure rather than whole-field time alone.
Reference dosimetry	Reference detector; calibration chain; dose-rate/DPP corrections; cross-calibration; measurement uncertainty	Dosimetric uncertainty should be smaller than the biological effect to be detected. At high DPP, ion recombination and detector response require specific evaluation.
Beam monitoring and QA	Pulse-/scan-resolved monitoring; machine logs; output stability; abnormal pulses; beam interruption; safety interlocks; patient-specific QA	Clinical studies require a traceable equipment-plan-patient closed loop.
Biological and model context	Species; age/sex; organ/tumor model; anesthesia and oxygenation; functional/histological endpoints; follow-up	These variables are effect modifiers and should not be treated as minor experimental details.
Comparator and statistics	CDR comparator; randomization/blinding; sample size; effect size and confidence interval; complete dose-response curve; negative results	Avoid defining a FLASH effect from significance at a single dose point. In animal tumor studies, “non-inferior” should not be used without a prespecified margin.

Reporting items are intended to prevent a single average dose-rate metric from substituting for a complete dose-space-time exposure description and to support cross-platform and inter-institutional reproducibility (17, 50–52).

These criteria convert a conceptual debate into three testable questions: how a given platform generates the intended temporal structure, whether that exposure can be measured accurately, and whether the measurement uncertainty is sufficiently small to resolve the biological effect being claimed.

## 3 Physical and dosimetric foundations of UHDR radiation

### 3.1 Radiation modalities and delivery platforms

Low-energy electron beams readily achieve high DPP and short total irradiation times and therefore dominated early FLASH research. Clinical linear accelerators can be modified through changes to the beam path, gun current, and pulse control to deliver small-animal UHDR irradiation, although penetration depth, field size, and dose uniformity limit deep-tissue applications. Reversible modifications improve experimental accessibility but also make output drift, mode switching, and pulse counting important components of routine quality assurance (19). Very-high-energy electrons (VHEE) offer greater penetration while retaining high dose-rate capability, although their depth-dose characteristics differ from the proton Bragg peak (20). Clinical VHEE translation will require coordinated solutions for high-energy acceleration, beam shaping, scanning speed, radiation shielding, and online monitoring (21).

Megavoltage X-rays are attractive for deep and large-volume irradiation, but bremsstrahlung conversion efficiency, target heat load, and pulsed output constrain the achievable DPP. Compact high-energy X-ray systems have produced varying degrees of tissue sparing in multi-organ mouse models and have shown that beam interruptions do not have identical effects across organs (22). These observations establish the technical feasibility of photon UHDR irradiation, but they do not justify the inference that every photon platform will produce a FLASH effect once an average dose rate of 40 Gy/s is reached.

Proton platforms can use transmission beams, scattering, or pencil-beam scanning. The design and *in vivo* validation of dedicated proton FLASH systems demonstrate the engineering feasibility of high-current beam modifications (23), and clinical proton systems adapted with scattering hardware and high-current monitoring have also been used in small-animal experiments (24). More recent cyclotron-based pencil-beam scanning platforms integrate scan control, temporal-structure recording, and biological validation, but trade-offs remain among Bragg-peak conformality, energy-layer switching speed, and local UHDR coverage (25). High-linear-energy-transfer (LET) radiation such as carbon ions differs from electrons and protons in track structure, oxygen enhancement, and complex DNA damage. Carbon-ion UHDR animal studies have shown that normal-tissue sparing and tumor control can coexist, although the evidence remains limited (26). Cross-modality comparisons of particle platforms must therefore report energy spectrum, depth-dose distribution, LET-related metrics, field size, and complete temporal structure in parallel (27), and temporal thresholds derived from low-LET platforms should not be extrapolated directly to high-LET irradiation (17, 28).

**Table 2** compares the major advantages, temporal characteristics, and translational constraints of these platforms. Platform differences are ultimately expressed not only through energy and penetration depth, but also through distinct combinations of DPP, scan timing, pulse intervals, and total delivery time. Radiation modality or nominal average dose rate therefore cannot substitute for a complete exposure description.

**Table 2:** Physical characteristics and clinical translational constraints of major UHDR delivery platforms.

Platform	Principal advantages and temporal characteristics	Major translational limitations	Interpretation of evidence/representative references
Low-energy electrons	High DPP, short pulses, and short total delivery times are readily achieved; platform modification is relatively mature	Limited penetration depth, field size, uniformity, and complex conformality; mode switching and output drift require dedicated QA	Well suited to superficial lesions, platform validation, and early human studies (18, 108).
VHEE	Greater penetration potential than conventional electrons while maintaining high dose rate	High-energy accelerators, beam shaping/scanning, shielding, and online monitoring remain systems-engineering challenges	Clear clinical potential, but complete equipment and QA infrastructures are still required (19, 20).
Megavoltage X-rays	Potential for deep and large-volume coverage; compatible with conventional photon workflows	Bremsstrahlung efficiency, target heat load, and achievable DPP are limiting; temporal structure varies substantially among devices	Animal-platform data are available, but reaching 40 Gy/s cannot be extrapolated to a universal FLASH effect (21, 31).
Protons	Transmission, scattering, and PBS delivery are possible; favorable depth-dose characteristics	Space-time coupling during PBS, local dose-rate definitions, energy-layer switching, and UHDR coverage create trade-offs	Engineering feasibility is established and FAST-01/02 provide human experience; comparative clinical benefit remains unproven (22–24, 109, 110).
Carbon ions/high-LET particles	High LET and distinct track structure provide unique biophysical conditions	Temporal thresholds from low-LET radiation cannot be directly extrapolated; evidence for clinical UHDR dosimetry and biological windows is limited	Animal data suggest coexistence of relative normal-tissue sparing and tumor control, requiring independent parameterized validation (16, 25–27).

### 3.2 Dose parameters, pulse structure, and fractionation effects

The biological effects of UHDR irradiation are jointly influenced by total dose, dose per fraction, average dose rate, DPP, intra-pulse dose rate, pulse width, repetition frequency, pulse number, pulse interval, total beam-on time, and macroscopic delivery time. Gastrointestinal studies indicate that, under specific experimental conditions, increasing either average dose rate or DPP can enhance normal-tissue sparing, but the effects of these two variables are not equivalent (29). When the same total dose is distributed across multiple pulse trains or the overall delivery time is prolonged by longer pulse intervals, intestinal sparing may diminish (30). Total delivery time is therefore an important determinant of the FLASH phenotype, but its role must be interpreted together with DPP, oxygen diffusion and reoxygenation, and radical reaction kinetics.

Fractionation is a major unresolved issue for clinical translation. Mouse intestinal studies show that reducing the dose per fraction can weaken or abolish normal-tissue sparing, indicating that a FLASH effect observed after a large single fraction cannot be extrapolated directly to conventional fractionation (31). Photon skin studies likewise demonstrate that acute injury and late tissue reactions are jointly influenced by total dose, dose rate, fraction size, and interfraction interval (32). Accordingly, 40 Gy/s should not be treated as an absolute boundary between the presence and absence of a FLASH effect. Quantitative studies across electron and photon platforms instead support evaluation of tissue-sparing magnitude within complete dose-effect and dose-rate-effect relationships rather than classification based on a single dose-rate metric (33).

Radiobiological parameters should also be considered when interpreting fractionation effects and developing quantitative models of the conditional FLASH effect. In particular, tissue- and tumor-specific α/β ratios may influence fractionation sensitivity and the balance between acute and late radiation responses (34, 35). Because these parameters may differ between experimental models and their human counterparts, especially for tumors, α/β assumptions should be specified where appropriate rather than extrapolated directly across species, tissues, or disease contexts (11, 12).

Coupling among these physical and radiobiological variables also complicates treatment planning. Several quantitative modeling approaches have therefore been explored for FLASH treatment planning, including joint dose–dose-rate optimization, FLASH-effectiveness models, and dose-modifying-factor approaches (36–38). Joint optimization of physical dose and dose rate can increase the fraction of organ-at-risk voxels satisfying a prespecified UHDR condition while preserving target-dose objectives, although such results remain model predictions (37). FLASH-effectiveness and dose-modifying-factor models can facilitate comparisons among scanning strategies and parameter settings, but their predictions depend strongly on assumptions regarding organ-specific biological parameters, dose-rate definitions, and effect thresholds (36). Related optimization frameworks can also express an assumed normal-tissue sparing effect as a “FLASH effective dose” for quantitative plan comparison (38). Until organ-specific effect sizes and underlying mechanisms are reproducibly validated, however, model-derived improvements cannot be equated with actual clinical benefit. Future modeling frameworks should therefore distinguish physical delivery descriptors from radiobiological inputs and explicitly report the assumptions and sources underlying parameters such as α/β ratios and dose-response relationships, particularly when parameters are extrapolated across species, tissues, tumor types, or acute and late response contexts (11, 12, 34).

Any parameter-effect relationship presupposes that the delivered parameters have been measured accurately. UHDR dosimetry and quality assurance are therefore not ancillary engineering considerations; they are foundational to biological comparison, multicenter reproducibility, and clinical safety.

### 3.3 Dosimetry and quality assurance

Accurate dose measurement under UHDR conditions is one of the key technical challenges for clinical translation. Conventional ionization chambers are susceptible to ion recombination and space-charge effects at high DPP, reducing charge-collection efficiency and causing under-response. The magnitude of this problem also depends on DPP, pulse width, electrode spacing, applied voltage, and electric-field strength (39). Ionization chambers optimized for UHDR conditions can improve charge collection by reducing electrode spacing and charge-drift distance and by optimizing the electric field (40). Dosimetry systems with relatively low dose-rate dependence, such as calorimeters and alanine dosimeters, can be used for reference dosimetry and dose transfer. Radiochromic film, owing to its high spatial resolution and lack of ion-recombination effects, is commonly used for dose-distribution verification in high-gradient regions and for cross-calibration among different dosimetry systems (41). Its response, however, cannot be assumed to be independent of dose rate and DPP under all UHDR conditions. Early electron-beam studies reported relatively stable EBT3 response within defined parameter ranges (42), whereas subsequent proton UHDR and high-DPP electron studies reported over-response of EBT3 or EBT-XD films under some irradiation conditions (43, 44). When radiochromic film is used for absolute dose or distribution verification under UHDR conditions, it should therefore be characterized independently for the specific radiation type, total dose, DPP, calibration method, and readout workflow and cross-validated with dosimeters based on different physical principles.

In addition to accurate measurement of total absorbed dose, temporal structure and instantaneous output must be monitored pulse by pulse. Current beam-monitoring technologies include ultra-thin parallel-plate ionization chambers, diamond detectors, scintillation detectors, semiconductor devices, and beam-current transformers. Ultra-thin parallel-plate chambers can reduce recombination losses by shortening the charge-drift path (45). Diamond detectors offer rapid response, small sensitive volumes, and high radiation tolerance, although linearity range, dose-rate dependence, and accumulated-dose effects must be validated for the specific device, beam condition, and application (46, 47). Beam-current transformers are non-interceptive monitors that, when coupled to high-speed acquisition, can record pulse waveforms, integrated charge, pulse number, and timing on a pulse-resolved basis and thereby identify extra pulses, missed pulses, or abnormal pulse amplitudes (44). Time-resolved scintillation imaging can simultaneously measure spatial dose distributions and microsecond-to-millisecond temporal structure during proton pencil-beam scanning and can validate log-file reconstruction and local dose-rate calculations (48). No single detector, however, can generally provide absolute dose accuracy, high spatial and temporal resolution, and a wide dynamic range simultaneously. A redundant dosimetric architecture based on detectors with different operating principles, together with cross-device and inter-institutional dose comparisons, is therefore a robust current strategy for improving the reliability of UHDR dose measurement and beam monitoring (49).

Once traceable reference dosimetry and time-resolved beam monitoring are established, clinical UHDR use requires an end-to-end quality-assurance program spanning acceptance and commissioning, routine operation, treatment-plan delivery, and patient-specific verification. In addition to total absorbed dose, QA should assess pulse or scan timing, total delivery time, output stability, field and depth-dose characteristics, patient positioning, treatment execution, machine logs, unplanned beam interruptions, and safety interlocks (50). A multi-institutional consensus for proton UHDR further recommends that machine-QA testing cover clinically relevant beam currents, energies, scanning modes, field sizes, and delivery-time structures whenever possible, with complete and traceable records of machine operation and irradiation (51). For electron UHDR systems, acceptance, commissioning, and routine QA should incorporate UHDR-specific quantities such as DPP, pulse width, repetition frequency, intra-pulse dose rate, and total irradiation time in addition to conventional electron-beam parameters (52, 53). Patient-specific QA is similarly evolving from absorbed-dose verification alone toward joint validation of spatial dose and time-resolved dose rate, although uniform evaluation methods and tolerances have not yet been established and should be validated for each platform within current frameworks (50, 51, 54). For millimeter-scale proton fields and high-dose-gradient regions, detector volume averaging, non-water equivalence, positioning uncertainty, and field-edge gradients must also be quantified; small-volume detectors, radiochromic film, and high-resolution imaging dosimetry may need to be combined (55). Overall, UHDR QA should form a closed-loop system anchored in traceable reference dosimetry, supported by pulse-resolved or time-resolved beam monitoring, and integrated across equipment-, plan-, and patient-specific verification to enable accurate, stable, and traceable delivery in FLASH clinical research and subsequent clinical translation.

Only after physical exposure and measurement uncertainty are constrained can biological effects be compared meaningfully across organs. The following sections therefore distinguish organ-specific positive, negative, and conditional evidence.

## 4 Tissue biological effects of UHDR radiation

### 4.1 Central nervous system effects

The central nervous system is one of the organ systems in which preclinical evidence for the FLASH effect is relatively concentrated and systematic. Early whole-brain electron UHDR studies showed reduced memory impairment in mice under specific dose and dose-rate conditions, whereas the protection weakened or disappeared at lower dose rates, suggesting dose-rate dependence of neuroprotection (56). Whole-brain synchrotron X-ray irradiation subsequently produced reductions in cognitive and histological injury, indicating that a similar normal-brain sparing phenotype can occur on different low-LET platforms (57). Longer-term studies found that, compared with CDR irradiation, UHDR irradiation reduced hippocampal dendritic-spine loss, neuroinflammation, and cognitive impairment and associated persistent neurocognitive benefit with a lower reactive-oxygen-species burden (58, 59). Reduced activation of reactive astrocytes and microglia further suggests that the effect may extend to longer-term glial responses and tissue homeostasis (60). Neuroprotection has also been reported in juvenile mice under UHDR conditions (61), and proton UHDR brain irradiation at the Bragg peak has produced normal-tissue sparing, extending the preclinical evidence across developmental stages and radiation modalities (62).

Existing brain studies include whole-brain and focal irradiation, single-fraction and hypofractionated schedules, and heterogeneous dose distributions, follow-up durations, and endpoints. However, much of the positive neurocognitive and histological sparing evidence derives from whole-brain irradiation in otherwise healthy rodents (56–62). Intracranial tumor-bearing models remain less extensively investigated, although neurocognitive sparing with preserved antitumor efficacy has been demonstrated in an orthotopic glioblastoma model (63). The central nervous system can therefore be regarded as one of the better-supported organ systems preclinically, but the strength and scope of this evidence vary across disease states and experimental designs, and the current evidence is insufficient to define a universal dose-rate, single-dose, or fractionation window for human brain irradiation.

### 4.2 Thoracic tissue effects

The lung was among the first organs in which normal-tissue sparing by FLASH irradiation was demonstrated. Early work showed that single-fraction UHDR electron irradiation reduced or delayed pulmonary fibrosis while maintaining tumor control comparable to that achieved with CDR irradiation (3). Subsequent studies implicated preservation of pulmonary epithelial progenitor cells and reduced radiation-induced cellular senescence in the attenuation of late lung injury (64). Lung sparing is not, however, an invariant property of UHDR irradiation; different single doses, dose rates, and fractionation schedules can produce different acute inflammatory, pulmonary-function, and fibrotic outcomes, indicating pronounced parameter dependence of the pulmonary FLASH phenotype (65).

Recent proteomic and single-cell transcriptomic studies add further layers of evidence. In a model using 3 Gy whole-body electron irradiation, the 200 Gy/s UHDR group showed less lung injury and collagen deposition at 3 months than the CDR group, together with attenuated CCT6b downregulation, changes in epithelial-mesenchymal-transition-related proteins, and enrichment of differential proteins associated with the mitochondrial respiratory chain (66). Because this study used a relatively low whole-body dose, the findings provide molecular clues to differential lung injury under UHDR conditions but cannot by themselves define a therapeutic pulmonary FLASH dose-dose-rate window. In another study using 17.8 Gy whole-thorax electron irradiation, UHDR irradiation caused less early impairment of lung function and, over 1–6 months of follow-up, less inflammatory infiltration, alveolar structural disruption, and collagen deposition than CDR irradiation; single-cell analysis also revealed early differences in immune-cell composition and inflammatory responses between dose-rate conditions (67). Further analysis of fibroblast subpopulations in the same single-cell dataset showed relative preservation of multiple fibroblast states at day 7 after FLASH irradiation. Mdk-positive fibroblasts exhibited stronger intercellular communication and transcriptional programs related to extracellular-matrix remodeling and structural organization, whereas CDR irradiation more prominently induced stress- and inflammation-related programs. These early stromal responses were associated with less later structural damage and fibrosis, although direct causality has not been established (68). Together, these findings extend the evidence for lung sparing across function, histopathology, immunity, and stromal remodeling, but differences among models in total dose, irradiated region, temporal structure, and endpoints still prevent direct comparison of parameter windows. Pulmonary studies therefore require complete dose-response relationships, functional assessment, and sufficiently long fibrosis follow-up to identify reproducible organ-specific conditions for protection.

Evidence for thoracic tissues other than the lung is more limited. In a mouse esophageal model, UHDR electron irradiation reduced acute histological injury and inflammation relative to CDR irradiation and was accompanied by less mitochondrial-associated damage (69). In a compact high-energy X-ray study, splitting the total dose into three deliveries separated by two 30-s pauses did not abolish normal-tissue sparing after whole-thorax irradiation, although protection was reduced compared with uninterrupted delivery. By contrast, segmented delivery during whole-abdominal irradiation did not retain the same degree of protection as single-sequence delivery (22). Because total dose and dose per segment differed between these organ models, the contrast cannot be attributed solely to organ specificity. Rather, it suggests that delivery interruption, dose per segment, and tissue context may jointly modify the FLASH phenotype. Temporal gaps introduced by clinical multi-field or scanning delivery should therefore be recorded and evaluated as part of the complete time structure rather than inferred from a global average dose rate.

Clinical thoracic irradiation can expose the lung, esophagus, heart, chest wall, and other normal tissues simultaneously. Endpoints from a single organ or a single time point are therefore insufficient to characterize overall normal-tissue benefit and long-term safety. Cross-organ large-animal and veterinary studies provide an important translational caution: even when acute reactions are mild, high single doses and large irradiated volumes may still be associated with severe late toxicity (4). Because these studies differ in irradiation site, total dose, and fractionation, the late injuries cannot simply be attributed to UHDR itself or extrapolated directly as risk estimates for thoracic organs. Their principal relevance is to underscore the necessity of independent long-term safety assessment during FLASH translation. Future thoracic FLASH studies should therefore use spatially and temporally comparable irradiation conditions, measure multi-organ function and histopathology, capture late fibrosis as well as vascular and bone injury, and assess normal-tissue sparing and tumor control in parallel.

### 4.3 Gastrointestinal effects

Gastrointestinal studies perhaps most clearly illustrate the conditional nature of the FLASH effect. Abdominal UHDR irradiation reduced gastrointestinal toxicity while maintaining tumor control in a mouse model of ovarian cancer (70), and other studies have reported preservation of intestinal crypts, enhanced stem-cell regeneration, and reduced acute toxicity (71). Negative evidence is equally clear: irradiation at 35 Gy/s failed to alleviate gastrointestinal syndrome or lymphopenia (72), and proton partial-abdominal irradiation did not produce tissue sparing, implicating irradiated volume and spatial dose gradients as potentially important sources of heterogeneity (73).

Inter-institutional electron FLASH comparisons have improved reproducibility of gastrointestinal findings by coordinating equipment, dosimetry, and biological endpoints (74). Parameter-focused studies further show that both average dose rate and DPP can modify the magnitude of protection (29). Intestinal benefit can decline markedly when total delivery time is prolonged or the dose is distributed over multiple pulse trains (30). More recent high-energy X-ray FLASH studies reported reduced intestinal inflammation and mitochondrial injury, whereas DNA double-strand breaks, apoptosis, and bone-marrow suppression did not improve to the same extent (75). This dissociation suggests that tissue sparing may arise from integrated preservation of barrier, metabolic, and regenerative networks rather than a uniform reduction in every cellular damage endpoint.

The gastrointestinal tract therefore cannot be classified simply as a FLASH-sensitive or FLASH-insensitive organ. Intestinal segment, irradiated volume, anesthesia, oxygenation, nutritional state, microbiome, single-fraction dose, and assessment time may all alter experimental outcomes. Future studies should avoid using short-term survival as a substitute for long-term barrier function, absorptive capacity, or fibrosis and should establish comparable parameter boundaries between positive and negative conditions.

### 4.4 Skin and hematopoietic system effects

Skin endpoints are visually accessible and well suited to construction of dose-response curves. Electron FLASH irradiation can reduce severe skin toxicity in mice (76), while proton pencil-beam scanning studies have quantified acute tissue sparing using dose-modifying factors (77). In a proton mouse-ear model, inflammation and tissue injury were reduced across different field dose-rate conditions, further demonstrating that average dose rate is not the sole determinant (78). Direct comparisons of electron and proton irradiation indicate that the magnitude of protection can differ between acute skin reactions and late fibrosis (79). Photon UHDR studies likewise show that total dose, dose rate, and fractionation or split-dose delivery jointly influence skin outcomes (32).

Cross-study comparison of skin effects should distinguish erythema, alopecia, ulceration, contraction, fibrosis, and muscle function rather than allowing a single acute score to substitute for long-term tissue function. In an immunocompetent melanoma model, FLASH and conventional irradiation achieved similar tumor suppression, but FLASH better preserved skin and muscle architecture and reduced persistent inflammation and metabolic disturbance (80). These findings strengthen the evidence for protection of long-term tissue homeostasis but still require replication across devices, dose schedules, and research centers.

Unlike skin, which primarily reflects local tissue response, the hematopoietic system is additionally shaped by exchange of circulating blood, organ perfusion, and systemic exposure, making its evidence more complex and warranting separate evaluation of dose-rate effects. Studies of human acute lymphoblastic leukemia and normal hematopoietic cells suggest that UHDR irradiation may relatively spare some normal hematopoietic progenitors while preserving leukemia-cell killing (81). In whole-animal models, however, circulating lymphocytes have not shown consistent benefit (72). Whether shortening irradiation time reduces the volume of circulating blood entering the field depends on organ perfusion, field size, and total delivery time and therefore cannot be regarded as a universal immune or hematopoietic protection mechanism.

### 4.5 Other organ effects and late toxicity

Beyond the central nervous system, lung, gastrointestinal tract, and skin, models of the liver and reproductive system are expanding the organ spectrum in which the FLASH effect is being investigated. In mice bearing breast tumors, electron UHDR irradiation reduced early liver injury, oxidative stress, and inflammation and better maintained metabolic homeostasis while preserving tumor-growth delay (82). This result suggests that normal-tissue sparing may involve integrated preservation of organ metabolism and functional state rather than local histological change alone. Because the study mainly assessed early outcomes, however, it does not establish whether protection persists into late phases or whether it alters chronic radiation-induced liver injury or fibrosis.

Reproductive-system studies likewise show pronounced dose and time dependence. After X-ray UHDR irradiation of mouse testes, less histological damage was observed at specific doses and assessment times, and subsequent work implicated GSTM3-regulated ferroptosis in the differential response (83). This provides a potential mechanistic link between redox imbalance and organ injury, but the current evidence remains dominated by histological and molecular endpoints. For the reproductive system, spermatogenesis, fertility, endocrine function, and genetic safety are of more direct clinical relevance; longer-term functional evaluation is therefore required to determine whether short-term tissue sparing translates into durable reproductive benefit.

Late toxicity has more direct safety implications for FLASH translation than acute tissue injury alone. Large-animal and companion-animal models provide important complementary evidence because of their tissue scale, clinically relevant irradiated volumes, and potential for long follow-up. In cats with squamous cell carcinoma of the nasal planum, some animals treated with a single 30-Gy UHDR fraction developed delayed maxillary osteonecrosis 9–15 months after irradiation. In mini-pigs exposed to single-fraction, large-field UHDR skin irradiation, severe late skin necrosis occurred in an irradiated-volume-dependent manner (4). These findings show that the absence of prominent acute toxicity does not guarantee long-term tissue safety; high single doses and large irradiated volumes may still exceed the tolerance of normal tissues for late injury.

These observations should not be interpreted as evidence that UHDR irradiation itself increases late toxicity. In the feline study, UHDR and CDR groups received different total doses and fractionation schedules, while the large-field mini-pig experiment lacked a same-dose, same-volume CDR comparator. The observed late injuries therefore cannot be attributed to dose-rate differences alone. Their more important translational implication is that the long-term safety of FLASH-RT remains constrained by total dose, fractionation, and irradiated volume and that acute normal-tissue sparing cannot replace independent validation of late safety.

Assessment of FLASH normal-tissue sparing should therefore include both acute and late outcomes. Future studies should record spatial dose, irradiated volume, fractionation, and temporal structure accurately and follow organ function, fibrosis, soft-tissue necrosis, and bone injury over clinically relevant intervals. Expansion of FLASH-RT will be justified only if normal-tissue sparing is reproducible under clinically relevant irradiation conditions and is not accompanied by unacceptable late toxicity.

### 4.6 Tumor control and microenvironmental effects

The central therapeutic premise of FLASH is that normal-tissue sparing must not come at the cost of tumor control. Across multiple transplanted and orthotopic tumor models, proton pencil-beam scanning UHDR irradiation has reduced normal-tissue injury while maintaining tumor control comparable to CDR irradiation (84). Overall, current evidence more strongly supports preservation of tumor control under UHDR conditions than a general additional tumoricidal effect. Some proton studies have reported greater tumor suppression together with immune-microenvironment changes such as increased T-cell infiltration, but this finding has not been reproduced consistently across tumor types, immune backgrounds, and irradiation conditions (85). Carbon-ion UHDR irradiation likewise maintained local tumor control in an osteosarcoma model while showing normal-tissue sparing and altered distant-metastasis outcomes (26). These observations suggest that tumor responses under high-LET conditions may involve immune and oxygen-dependent processes that differ from those under low-LET irradiation, but the current evidence remains too limited for a unified mechanistic interpretation.

Cell-level studies alone do not adequately explain the differential response of normal tissue and tumor. In normal human lung fibroblasts, proton UHDR irradiation had limited effects on clonogenic survival and early γ-H2AX responses but reduced long-term senescence and TGF-β1 levels (86), suggesting that dose-rate differences may influence sustained cellular stress and late-injury pathways more strongly than initial cell killing. A further multilevel study found relative preservation of mitochondrial structure and function in nonmalignant pancreatic cells after UHDR irradiation, whereas pancreatic cancer cells did not show equivalent protection. In tumor-bearing animals, UHDR and CDR irradiation produced comparable tumor-growth delay, and the survival advantage with UHDR was attributed primarily to reduced normal-tissue toxicity rather than stronger tumor control (87). These findings indicate that differences in cell-intrinsic radiosensitivity alone are unlikely to explain selective normal-tissue sparing by FLASH.

The tissue microenvironment may represent a critical level at which this differential response emerges. Changes in perfusion, oxygenation, immune-cell infiltration, and inflammatory responses have been observed, but causal relationships between these changes and the FLASH phenotype have not been established (88). Many UHDR experiments also use a large single fraction or a small number of large fractions; observed immune and microenvironmental changes may therefore contain both UHDR temporal-structure effects and biological effects of hypofractionation itself. To isolate UHDR-specific effects, comparative studies should match total dose, radiation quality, spatial dose distribution, and irradiated volume as closely as possible, treat temporal structure as the main experimental variable, and assess tumor control and adjacent normal-tissue injury within the same framework. Tumor endpoints should incorporate an explicit time dimension, such as tumor-growth delay, local control, or dose-response relationships, rather than relying solely on tumor volume at a single time point. An additional translational caveat is that small-animal tumor models may differ from their human counterparts in genetic heterogeneity, cell kinetics, stromal and immune context, and the extent and spatial distribution of hypoxia (89). Potential differences in radiobiological characteristics, including fractionation sensitivity and α/β ratios, further limit the direct extrapolation of tumor responses from experimental models to human tumors (11, 12). Consequently, preservation of tumor control in a particular small-animal model should not be assumed to predict an equivalent tumor response in humans.

**Table 3** condenses the major organ and tumor findings into a comparative evidence matrix. Relative normal-tissue sparing with preserved tumor control is the core therapeutic phenotype that any FLASH mechanism must explain. Mechanistic models must therefore address not only why normal tissue may be protected, but also why that protection does not extend to tumors to the same degree.

**Table 3:** Comparative evidence matrix for major organ systems and tumors.

System	Supportive evidence	Negative/conditional evidence and major modifiers	Translational interpretation
Central nervous system	Multiple electron, X-ray, and proton models report reduced cognitive, inflammatory, or histological injury (56–62).	Whole-brain versus focal exposure, dose, fractionation, and follow-up vary substantially.	Preclinical evidence is relatively concentrated; no unified human parameter window has been established.
Lung/thorax	Multilevel findings in fibrosis, function, inflammation, and stromal remodeling support conditional sparing (64, 66–68).	Dose/fractionation dependence is pronounced; large-animal late toxicity highlights volume and long-term risk (5, 65).	Multi-organ function and long-term fibrosis, vascular, and bone endpoints are needed.
Gastrointestinal tract	Selected electron/photon models show crypt preservation, regeneration, and reduced acute toxicity (69, 70, 73, 74).	Negative findings at 35 Gy/s and after proton partial-abdominal irradiation; DPP, total delivery time, volume, oxygenation, and other factors are influential (28, 29, 71, 72).	This system most clearly illustrates conditionality; organ-specific dose-response boundaries should be established.
Skin	Electron, proton, and selected photon models show reductions in acute or late injury (31, 75–78).	Protection differs between acute and late endpoints; total dose and fractionation/split delivery are important.	Well suited to dose-response quantification and comparative studies of superficial human lesions.
Hematopoietic/circulating system	Relative sparing signals have been reported for some normal hematopoietic progenitor cells (80).	Circulating lymphocytes are not consistently spared in whole-animal models; perfusion, field size, and total delivery time are influential (71).	Faster irradiation should not be equated universally with immune or hematopoietic protection.
Other organs and late toxicity	Liver and reproductive-system models broaden the organ spectrum (81, 82).	Most studies emphasize early endpoints; cat and mini-pig data show that severe late injury can occur after large-volume and high-single-dose exposure (5).	Long-term organ function and late toxicity are non-substitutable safety levels.
Tumor	Multiple models generally support preserved tumor control under UHDR conditions (3, 25, 83, 85).	Additional tumor sensitization and immune advantages are not consistently reproduced; spatial dose, total dose, and volume must be matched (4, 86).	Animal studies should describe preserved/comparable tumor control; formal non-inferiority margins belong in prespecified clinical trials.

“Supportive” evidence denotes directionally consistent findings under specific models and parameters, not a universal cross-platform conclusion.

## 5 Molecular mechanisms and biological hypotheses underlying the FLASH effect

Any explanatory model of the FLASH effect must satisfy several key evidentiary constraints simultaneously: it should explain dependence on the ultrashort temporal structure of UHDR delivery, account for differential responses between normal tissue and tumor, accommodate heterogeneity in effect magnitude across organs, radiation qualities, and irradiation parameters, and explain why both positive and negative results occur. Current evidence does not support a single molecular event as a sufficient explanation of the FLASH phenotype. A more consistent interpretation is that early radiation-chemical differences are amplified or modified through successive cellular and tissue-level responses, ultimately producing normal-tissue sparing that is conditional on the exposure and biological context.

### 5.1 Oxygen dynamics and radiation chemistry

Oxygen-related mechanisms were among the earliest hypotheses proposed to explain the FLASH effect. The central idea is that UHDR irradiation may consume local dissolved oxygen rapidly enough to precede diffusion and blood-borne replenishment, transiently reducing oxygen fixation. *In vitro* studies show that biological differences between UHDR and CDR irradiation can depend on oxygen concentration, becoming more apparent under certain low-to-intermediate oxygen conditions and attenuating at extremely low or relatively high oxygen levels (90). Thus, within oxygen-dependent models, a FLASH-associated sparing difference may be more likely to emerge under pre-existing low-to-intermediate oxygenation than in well-oxygenated conditions, although low oxygen is not a universal requirement and the relevant oxygen window remains model dependent (90–92). Oxygenation is also directly coupled to radiation chemistry: molecular oxygen reacts with radiation-induced carbon-centered radicals to form peroxyl radicals, while competing radical–radical reactions can alter peroxyl-radical formation and the yields and persistence of downstream reactive species (93, 94). Quantitative analyses further indicate that baseline oxygen partial pressure, total dose, and oxygen consumption per unit dose can influence changes in oxygen enhancement (91), and computational models similarly support joint modulation of potential protection by oxygenation and irradiation dose (95).

Current evidence, however, does not support complete oxygen depletion as a universal explanation of the FLASH effect. Chemical-track simulations indicate that, across most physiological oxygenation states and clinically relevant dose ranges, radiation-induced bulk oxygen consumption is generally insufficient to create the degree of complete hypoxia needed to account for the magnitude of tissue sparing observed experimentally (96). Time-resolved *in vivo* measurements do show transient decreases in oxygen after UHDR irradiation, with magnitude dependent on baseline oxygenation and dose (97). Conversely, water-system experiments using photon, proton, and carbon-ion irradiation have not demonstrated complete oxygen depletion at clinically relevant doses (98). The latter findings reflect radiation chemistry in simplified systems and cannot substitute directly for oxygen dynamics in living tissues, where perfusion, diffusion, and metabolism interact.

A more defensible current interpretation is therefore that transient oxygen changes within local tissue microenvironments or at subcellular scales may contribute to the FLASH phenotype but are insufficient as a stand-alone mechanism. Extrapolation of radiation-chemical models to tissue must account for the complexity of biological media. Inferring tissue effects solely from oxygen consumption or hydrogen-peroxide yield in pure water risks overlooking competing reactions and regulatory processes involving proteins, lipids, metal ions, and endogenous antioxidant systems (94). Future studies should couple time-resolved measurements of oxygen dynamics and radiation chemistry with organelle function, tissue injury, and long-term functional outcomes to determine whether early oxygen transients propagate across time scales and contribute causally to stable normal-tissue sparing.

### 5.2 Radical reactions and redox homeostasis

A related mechanistic framework emphasizes altered radical reaction kinetics under UHDR conditions. Extremely high instantaneous radical density can increase the probability of radical-radical recombination, thereby altering formation of peroxyl radicals and downstream oxidation products. Kinetic models identify peroxyl-radical recombination as one radiation-chemical process that may contribute to differences between UHDR and CDR irradiation (93). This process is also modulated by radical production rate, local oxygen concentration, and the redox-buffering capacity of the biological system. Differences between normal tissues and tumors in antioxidant reserves, lipid composition, and oxidative metabolism may therefore modify the biological consequences of early radiation chemistry (1).

Accordingly, the existing evidence is better captured by spatiotemporal changes in redox homeostasis than by the broad claim that FLASH simply reduces the total amount of reactive oxygen species (ROS). UHDR irradiation may alter the timing, local distribution, and molecular composition of ROS rather than uniformly suppressing all oxidative signals (99). Nrf2-associated antioxidant responses, the glutathione system, iron metabolism, and lipid peroxidation may contribute to this modulation, but most evidence remains associative, ex vivo, or model-based. Direct causal evidence linking early radical dynamics to final functional injury in intact tissues remains limited.

Ferroptosis may represent one downstream link between redox imbalance and tissue injury. Testicular UHDR studies show that GSTM3-related regulation and interventions targeting ferroptosis can alter tissue injury across dose-rate conditions, providing initial interventional evidence that this pathway contributes to FLASH-associated differences (83). At present, however, the finding derives mainly from a single organ and specific irradiation conditions. Its generalizability across organs and radiation modalities, and its influence on long-term functional outcomes, remain to be established.

### 5.3 DNA damage and cell fate

Whether UHDR irradiation protects normal tissue by reducing initial DNA damage remains unresolved. Comet assays indicate that UHDR and CDR irradiation can produce different spectra of DNA damage under specific experimental conditions and may alter some forms of oxidative damage and their subsequent processing (100). In normal cells, proton UHDR irradiation has relatively limited effects on clonogenic survival and early γ-H2AX responses but reduces longer-term cellular senescence (86). Because γ-H2AX signals depend on measurement time, cell cycle, chromatin state, and repair processes, changes in this marker cannot be interpreted directly as changes in the number of initial DNA double-strand breaks.

Other genetic-damage endpoints further illustrate the complexity. Studies of chromosomal rearrangements have not demonstrated stable, specific protection from radiation-induced rearrangements under UHDR irradiation (101), whereas human peripheral-blood lymphocytes exposed under particular electron UHDR conditions showed fewer stable and unstable chromosomal aberrations (102). The incomplete agreement across experiments indicates that dose-rate effects on DNA and chromosomal damage are strongly dependent on cell type, irradiation conditions, and endpoint and cannot yet be summarized as a universal reduction in genetic damage under UHDR irradiation.

Current evidence therefore does not support a simple model in which FLASH tissue sparing results from a generalized reduction in initial DNA breaks. More informative questions may concern the chemical nature and spatial distribution of damage, repair kinetics, and subsequent differences in cell death, senescence, and damage-associated signaling. Because these cell-autonomous changes are still insufficient to explain the complete organ-level FLASH phenotype, DNA damage should be evaluated within higher-order processes that include mitochondrial homeostasis, inflammation, and tissue repair.

### 5.4 Mitochondrial and metabolic homeostasis

Mitochondria sit at the intersection of radiation-induced oxidative stress, energy metabolism, cell death, and innate immune signaling and may therefore connect early radiation-chemical differences to later cellular and tissue responses. In normal fibroblasts, mitochondrial alterations associated with hypoxia or cytochrome-c deficiency enhance relative radioresistance under UHDR conditions and modify post-irradiation cell death (103), suggesting that mitochondrial state can modulate dose-rate effects.

Models closer to the tissue level provide additional support. Human intestinal organoids show distinct transcriptional and structural responses after UHDR versus CDR irradiation, offering a system with greater tissue organization than two-dimensional culture for evaluating epithelial regeneration, metabolism, and barrier-related responses. Because organoids lack intact perfusion and immune environments, however, their findings require validation in immunocompetent animal models (104). Multilevel studies further show relative preservation of mitochondrial structure and metabolic function in nonmalignant pancreatic cells under UHDR irradiation, without equivalent protection in pancreatic cancer cells; *in vivo*, survival benefit was associated primarily with lower normal-tissue toxicity rather than additional tumor-control enhancement (87). High-energy X-ray gastrointestinal studies have also associated normal-tissue sparing with less mitochondrial injury (75), and liver studies suggest that maintenance of metabolic homeostasis may contribute to early tissue protection (82).

Evidence for selective mitochondrial preservation is increasing, but its position in the causal chain is not yet defined. Mitochondrial preservation could be a downstream consequence of early radiation-chemical differences, or mitochondria could independently influence tissue outcomes through ROS amplification, metabolic reprogramming, and inflammatory signaling. Distinguishing these possibilities will require time-resolved measurements, organelle-specific perturbation, and reversibility or rescue experiments.

### 5.5 Immune, vascular, and tissue-microenvironment effects

The extremely short delivery time of UHDR irradiation could, under selected conditions, reduce the volume of circulating blood entering the irradiation field during exposure, but the actual effect depends strongly on irradiated volume, organ perfusion, and total delivery time. Brain studies have reported attenuated glial responses and inflammatory signals, whereas some whole-animal models have not shown lymphocyte sparing; some tumor models, in contrast, report increased T-cell infiltration or other immune-microenvironment changes (59, 72, 85). Dose, irradiated volume, tumor burden, and large-fraction effects are all potential confounders, and changes in individual cytokines or immune-cell proportions are therefore insufficient to establish a UHDR-specific immune mechanism.

Single-cell studies of lung tissue further suggest that UHDR and CDR irradiation can induce distinct early immune and repair responses. In a secondary stromal-cell analysis of the same lung single-cell dataset, multiple fibroblast states were relatively preserved at day 7 after UHDR irradiation. Mdk-positive fibroblasts showed stronger cell-cell communication and transcriptional features related to extracellular-matrix remodeling and structural organization, whereas CDR irradiation more prominently induced stress- and inflammation-related programs. These early stromal patterns were associated with better later preservation of lung architecture and less fibrosis (68). The current evidence is nevertheless based mainly on transcriptomics, inferred cell-cell communication, and histological association and does not establish a causal role for a specific fibroblast state or signaling pathway in normal-tissue sparing.

At the tissue scale, vascular function, immune cells, extracellular matrix, and regenerative capacity may jointly regulate differential responses of normal tissue and tumor to UHDR irradiation and may help explain why isolated cell systems often fail to reproduce the full FLASH phenotype (9). Future studies should match total dose and spatial dose distribution, treat temporal structure as the primary experimental variable, and integrate spatial omics, multiplex immune imaging, vascular-function measurements, and lineage tracing to distinguish accompanying changes from effect modifiers and mechanistically decisive nodes.

### 5.6 Multiscale coupling and causal validation

The available evidence favors a multiscale coupling model across time rather than a single-mechanism explanation. Oxygen and radical dynamics on microsecond-to-millisecond time scales may first alter the chemical environment of radiation damage, which may then influence cell fate through membrane-lipid oxidation, the nature of DNA damage, redox regulation, and mitochondrial homeostasis. Over hours to months, these early differences may be expressed as altered inflammatory and immune responses, stem-cell and epithelial regeneration, vascular function, tissue remodeling, and fibrosis (105). Baseline oxygenation, antioxidant capacity, turnover rate, and matrix architecture differ among tissues, so the relative contribution of each mechanistic step may vary by organ, radiation modality, and irradiation condition. Lung proteomic and single-cell studies extend this evidence chain to mitochondrial and epithelial-mesenchymal-transition homeostasis, immune-epithelial repair networks, and fibroblast-extracellular-matrix remodeling (65–67), but these findings remain largely associative evidence from a single organ and are insufficient to establish a complete causal chain. **Figure 2** summarizes this candidate multiscale causal pathway and its principal effect modifiers.

**Figure 2:**
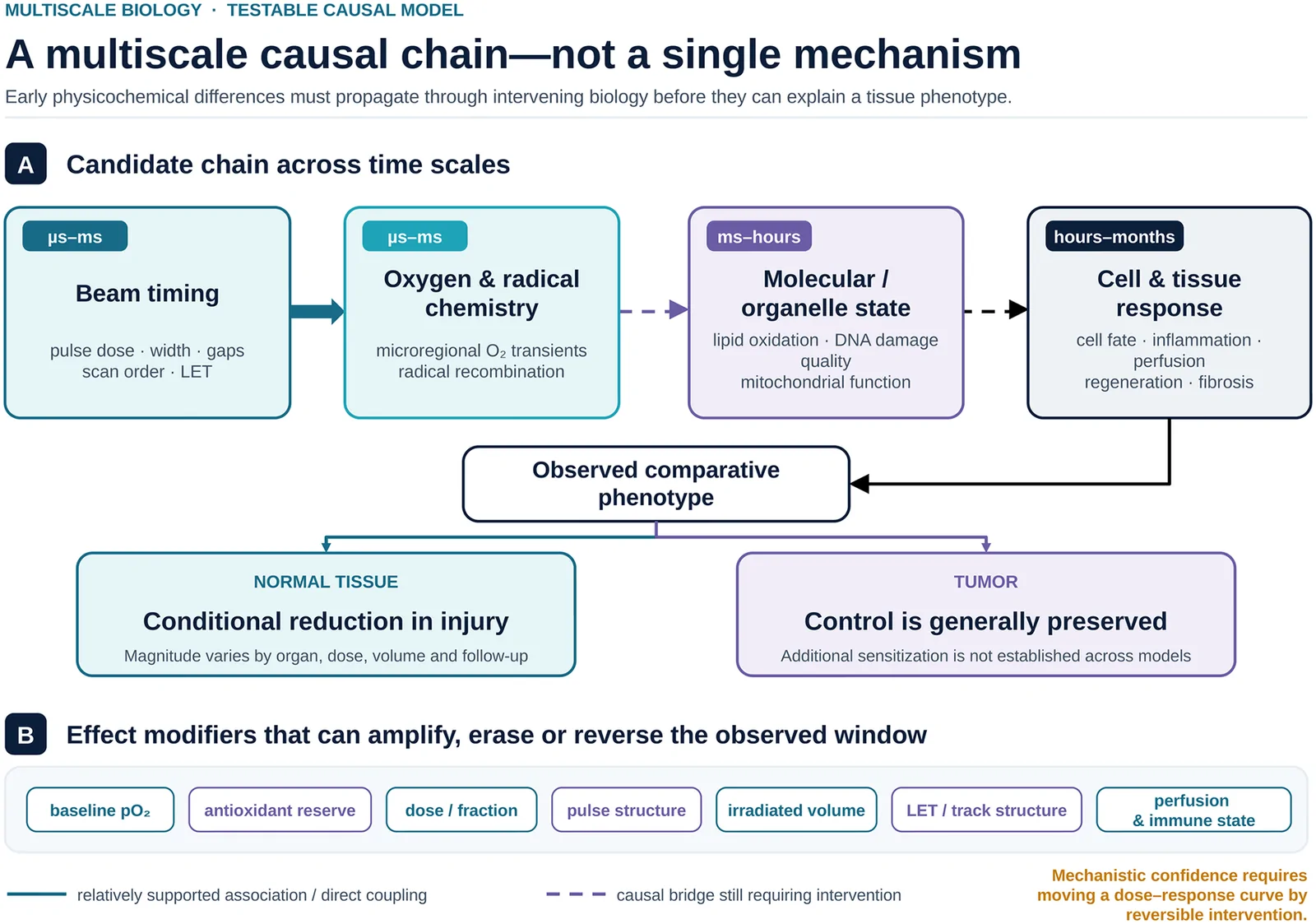
Candidate multiscale causal chain and effect modifiers of the FLASH phenotype. **(A)** The candidate causal chain extends from beam timing and oxygen/radical chemistry on microsecond-to-millisecond time scales, through molecular and organelle states, to cellular and tissue responses, ultimately producing conditional reduction of normal-tissue injury with generally preserved tumor control. **(B)** Baseline oxygen partial pressure, antioxidant reserve, dose per fraction, pulse structure, irradiated volume, LET/track structure, perfusion, and immune state may amplify, attenuate, or reverse the observed window. Solid connections represent relatively direct or better-supported associations; dashed connections denote causal bridges that still require interventional testing. The figure is a conceptual synthesis of the evidence reviewed here rather than an established complete mechanistic chain (87, 88, 91, 94–96, 98, 102, 104, 105).

Mechanistic research should therefore move beyond cataloguing associated molecules and pathways toward interventional and reproducible causal tests. An ideal design would hold total dose and spatial dose distribution constant while systematically altering pulse structure, measure early oxygen and radical dynamics whenever feasible, and then use genetic or pharmacological perturbations to test candidate nodes such as mitochondrial homeostasis, antioxidant responses, ferroptosis, immune inflammation, and stromal remodeling (10). If perturbation of a given node reproducibly shifts the normal-tissue dose-response relationship while tumor control is assessed in parallel, the evidence for causality would be substantially stronger. Consistent directional results across platforms and organs would then be required before broader mechanistic generalization.

Incomplete mechanistic understanding is not an absolute prerequisite for early clinical feasibility studies, but it limits the reliability of parameter extrapolation, patient stratification, and mechanism-guided treatment optimization. While mechanistic evidence remains incomplete, clinical translation should therefore rely even more heavily on traceable physical exposure, rigorous dosimetry and QA, prespecified normal-tissue and tumor endpoints, and appropriate comparative designs to reduce interpretive risk arising from mechanistic uncertainty.

## 6 Clinical translation of FLASH-RT

### 6.1 Human evidence

In 2019, the first patient treated with electron FLASH-RT received 15 Gy to a localized cutaneous T-cell lymphoma lesion over 90 ms, with a complete short-term tumor response and mild local skin toxicity (106). This case provided the first demonstration that UHDR irradiation could be delivered technically in a human patient. A single case, however, cannot exclude confounding by spatial dose distribution, lesion characteristics, or individual variability, cannot establish comparative normal-tissue sparing relative to CDR irradiation, and cannot adequately evaluate late toxicity. Its primary significance is therefore proof of clinical feasibility rather than confirmation of a human FLASH effect.

Electron FLASH subsequently entered prospective clinical evaluation. IMPulse was a phase I dose-escalation study in patients with progressive cutaneous melanoma metastases, using single-fraction 9-MeV electron FLASH irradiation at 22–28 Gy and dose rates above 200 Gy/s. Ten patients with 15 lesions were treated, and the maximum tolerated dose was not reached within the prespecified 4-week dose-limiting-toxicity window (107). Slow accrual prevented completion of the originally planned escalation to higher dose levels, and the primary endpoints emphasized short-term toxicity. The findings therefore support near-term tolerability within the tested dose range but do not establish durable normal-tissue protection or superior therapeutic gain over CDR irradiation. In parallel, the Flash-Skin I study implemented electron UHDR treatment in humans using a modified conventional clinical C-arm linear accelerator and verified patient-level dose and pulse delivery with multiple dosimetric systems (108). This work further demonstrates that existing clinical accelerators can support traceable human UHDR delivery after systematic commissioning, redundant dosimetric validation, and dedicated QA; however, its main contribution is technical implementation and quality assurance rather than evidence of FLASH-mediated normal-tissue sparing.

Human proton FLASH evidence currently derives mainly from the FAST series. FAST-01 used transmission proton beams to deliver a single 8-Gy fraction to 12 painful extremity bone metastases in 10 patients. No serious device- or delivery-related safety events were reported, and pain response and adverse-event patterns were broadly consistent with historical experience after conventional single-fraction 8-Gy palliative radiotherapy (109). FAST-02 extended treatment to nonvertebral bone metastases near critical thoracic normal structures and likewise supported the clinical feasibility of proton UHDR treatment, with acceptable preliminary safety and analgesic effects (110). This progression shows that proton UHDR delivery can move from relatively simple extremity lesions to anatomy closer to organs at risk. Both FAST-01 and FAST-02, however, were small, single-arm studies without concurrent CDR controls, and currently available follow-up remains limited for late toxicity. Their evidentiary contribution should therefore be restricted to workflow feasibility, preliminary safety, and palliative efficacy signals; they do not establish normal-tissue sparing, superior tumor control, or improved therapeutic ratio relative to CDR irradiation.

Taken together, human studies consistently indicate that selected electron and proton platforms can deliver UHDR irradiation to patients according to protocol and provide evidence of clinical feasibility and preliminary safety. They have not yet answered the central comparative clinical question for FLASH-RT: whether, under comparable total dose, spatial dose distribution, and other treatment conditions, UHDR irradiation can reproducibly reduce normal-tissue toxicity without compromising tumor control. Human evidence should therefore currently be classified as demonstrating clinical-delivery feasibility and early safety rather than establishing a clinical FLASH effect. Normal-tissue sparing relative to CDR irradiation and any eventual gain in therapeutic ratio require comparative studies with appropriate concurrent controls, adequate follow-up, and prespecified tumor-control endpoints. LANCE (NCT05724875) is a randomized phase II selection trial directly comparing FLASH-RT with CDR irradiation for localized cutaneous squamous cell carcinoma or basal cell carcinoma (111, 112). A phase I protocol registered as ChiCTR2400080935 has also been published to evaluate the safety and feasibility of fractionated electron FLASH-RT for localized superficial skin tumors (113). **Figure 3** summarizes the published human evidence and presents a staged translational pathway from traceable physical exposure and reproducible organ-specific sparing windows through clinical-grade delivery to comparative clinical benefit.

**Figure 3:**
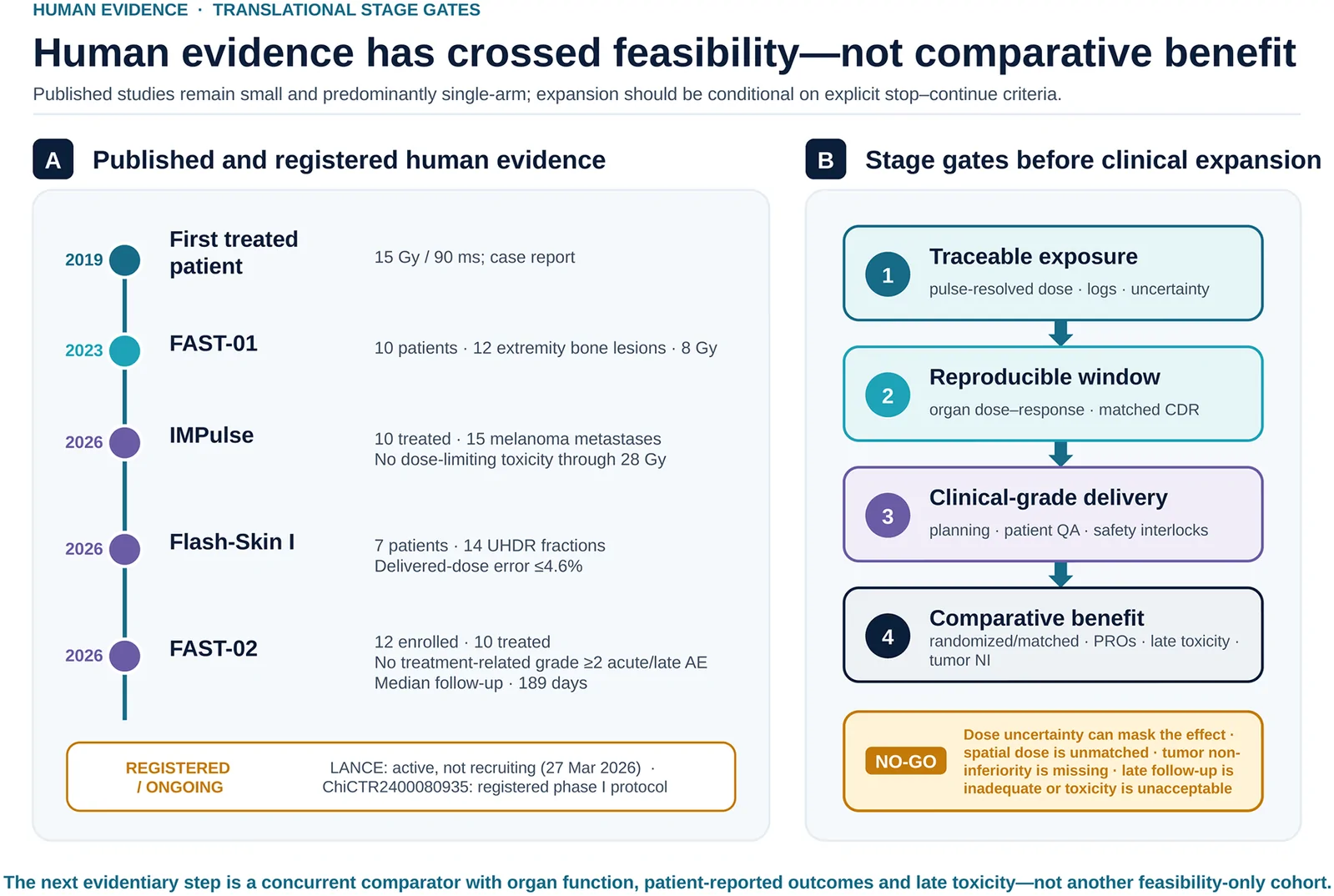
Human UHDR/FLASH-RT evidence and stage gates for clinical expansion. **(A)** The first treated patient, FAST-01, IMPulse, Flash-Skin I, and FAST-02 collectively support the technical feasibility of human UHDR delivery and provide varying degrees of near-term safety and implementation evidence. These studies remain small and predominantly single-arm and therefore cannot independently demonstrate comparative normal-tissue sparing. IMPulse enrolled 11 patients and treated 10 patients with 15 lesions; no dose-limiting toxicity was observed within the prespecified 4-week window. FAST-02 enrolled 12 patients and treated 10, with a median follow-up of 189 days. LANCE (NCT05724875) is a randomized trial comparing FLASH and CDR irradiation; ClinicalTrials.gov listed the study as active, not recruiting on March 27, 2026. A phase I protocol has been published for ChiCTR2400080935. **(B)** Clinical expansion should sequentially cross stage gates for traceable exposure, a reproducible organ-specific window, clinical-grade delivery, and comparative benefit. Excessive dosimetric uncertainty, spatial dose mismatch, absence of an adequate tumor-control framework, insufficient late follow-up, or unacceptable toxicity should trigger a no-go decision (107–111, 113–115).

### 6.2 Equipment, planning, and delivery constraints

Translation of FLASH-RT from experimental platforms into clinical practice requires more than the ability to generate a high nominal dose rate. Clinically relevant fields, depths, and patient geometries must simultaneously support dose conformality, temporal-structure control, beam stability, online monitoring, and patient-level dose verification. Proton pencil-beam scanning can improve spatial conformality for complex targets, but scan trajectories, local re-irradiation, and energy-layer switching alter the dose-accumulation time of individual voxels, making plan-level average dose rate a poor representation of actual tissue exposure (15). Electron platforms more readily achieve high DPP and short total delivery times, but their clinical use remains constrained by penetration depth, field size, and conformality for complex field shapes. Reaching a nominal UHDR threshold is therefore insufficient to define a clinical FLASH exposure; the local voxel dose-accumulation process and its spatial heterogeneity must be retained for cross-platform biological comparison and patient exposure characterization.

Device accessibility and clinical scalability also shape the translational pathway. Reversible modification of existing clinical accelerators and progressively standardized electron UHDR platforms can lower the technical barrier to clinical and translational research (114). A nominal average dose rate of 40 Gy/s, however, does not establish clinical-grade output stability, dose monitoring, redundant verification, or safe interlocking under abnormal conditions. Human electron UHDR studies further show that formal clinical use requires multilevel verification of output stability, pulse number, and the absorbed dose actually delivered to the patient before and during treatment (108). Commissioning and QA are therefore integral to the definition and interpretation of the patient’s physical exposure, not auxiliary procedures in a clinical study.

Treatment-plan evaluation likewise cannot be limited to global or field-averaged dose rate. For scanning beams and complex multi-field plans, spatial dose, local dose-accumulation time, and dose-rate distributions within targets and organs at risk should be described together, and the selected dose-rate definition and calculation window should be explicit. The fraction of voxels meeting a prespecified dose-rate or delivery-time condition can serve as a physical exposure descriptor for plan comparison (38), but until organ-specific biological thresholds are reproducibly validated it should not be interpreted directly as a probability of normal-tissue sparing. Respiratory and organ motion, multi-field superposition, gating, energy-layer switching, gantry transitions, and unplanned beam interruptions can all reshape local temporal structure. Clinical planning must therefore expand from conventional dose-space optimization to integrated dose-space-time control.

Correspondingly, clinical UHDR protocols must progress from the task of demonstrating that an experimental platform “can irradiate” to that of establishing dose delivery that is traceable, reproducible, and auditable. During clinical operation, output stability after machine warm-up, DPP, pulse number and width, repetition frequency, abnormal pulses, and monitoring-chain response require continued verification (53). Proton UHDR machine QA should cover high beam currents, scan modes, energies, and clinically relevant temporal structures (51), whereas electron UHDR systems require UHDR-specific metrics such as DPP, intra-pulse dose rate, and total irradiation time in addition to conventional acceptance and QA tests (52). Equipment development, treatment planning, reference dosimetry, time-resolved beam monitoring, and patient-specific QA must therefore operate as a closed loop. A systematic error at any point can obscure a true biological difference or cause a physical-delivery difference to be misinterpreted as a FLASH effect.

### 6.3 Clinical trial design and translational pathway

FLASH-RT trials must first distinguish three different claims: that treatment is effective, that treatment is safe, and that comparative normal-tissue sparing exists. Pain relief, tumor shrinkage, or local control can assess antitumor or palliative efficacy but cannot serve as surrogates for normal-tissue protection; conversely, mild short-term skin reactions cannot substitute for assessment of late tissue safety. Clinical endpoints should be prespecified according to the irradiated organ, therapeutic intent, and expected survival and should incorporate organ function, patient-reported outcomes, standardized toxicity grading, and organ-specific late injury such as fibrosis, soft-tissue or bone necrosis, neurocognitive decline, and vascular damage (2). Curative treatment and populations expected to survive long term require follow-up that captures the relevant latency of major late toxicities and, where appropriate, long-term risks such as second malignancies.

Early clinical studies may reasonably prioritize superficial or palliative indications with clearly bounded dosimetry, easily localized lesions, relatively low risk of catastrophic unexpected toxicity, and readily observed clinical endpoints in order to validate devices, workflows, and near-term safety. Once the objective shifts from feasibility and safety to demonstrating FLASH normal-tissue sparing or an improved therapeutic ratio, however, historical controls are generally insufficient. Subsequent studies should preferentially include concurrent CDR comparators and, when feasible, randomized designs. In suitable patients with multiple similar superficial lesions, within-patient paired strategies may also reduce interindividual heterogeneity. Across dose-rate groups, total dose, fractionation, radiation quality, spatial dose distribution, and irradiated volume should be matched as closely as possible so that temporal structure remains the principal experimental variable and technological, planning, or patient differences are not misclassified as a FLASH effect (115).

Clinical translation also requires a patient-level evidence chain connecting machine output to clinical outcome. Pulse- or scan-resolved machine logs, reference and *in vivo* dosimetry, voxel-level dose and dose rate, complete delivery-time structure, treatment imaging, biospecimens, and acute and long-term clinical outcomes should be linked through standardized data structures. QA frameworks should be incorporated at the protocol-design stage rather than added only during interpretation of results (50), and definitions, recording requirements, and reporting standards for UHDR parameters should be prespecified in protocols, case-report forms, and data dictionaries (18). Reliable cross-device, cross-modality, and multicenter exposure-outcome analyses will be possible only when centers can accurately reconstruct the physical exposure actually experienced by each patient.

FLASH-RT development is therefore better suited to prespecified stage-gated continue/stop criteria than to indication expansion based on a single average dose-rate threshold. At the physical level, absorbed dose and complete temporal structure must be demonstrably traceable under clinically relevant conditions, with measurement and delivery uncertainty sufficiently smaller than the biological effect the study is designed to detect. At the biological level, reproducible normal-tissue sparing must be shown at clinically relevant total doses, irradiated volumes, and functional endpoints. At the oncological level, tumor control must at minimum be shown not to be worse than that of the relevant CDR treatment. At the safety level, unacceptable late toxicity must be excluded over a follow-up interval appropriate to the target organ. Expansion to larger irradiated volumes, greater organ-at-risk exposure, more complex fractionation, or curative-intent treatment should occur only after the prespecified validation criteria at the preceding level have been met.

The central translational question is therefore no longer simply whether UHDR delivery can be achieved, but which measurable and reproducible physical-biological conditions justify comparative clinical testing and how appropriate control designs and long-term follow-up can demonstrate a clinically meaningful therapeutic gain at acceptable risk. This transition is the critical step required for FLASH-RT to move from technical feasibility studies toward a predictable, testable, and auditable clinical strategy.

## 7 Discussion and outlook

UHDR pulsed irradiation makes temporal structure an explicit therapeutic variable that must be characterized and optimized alongside spatial dose distribution, and selected preclinical models have shown differential responses in which normal-tissue injury is reduced without loss of tumor control. Across the current evidence base, UHDR as a physical delivery state should remain strictly distinct from the FLASH effect as a comparative biological phenotype. The latter is not an intrinsic property that necessarily appears once a nominal dose rate is exceeded; it is more plausibly a conditional phenotype jointly determined by total dose, DPP, pulse structure, radiation quality, irradiated volume, tissue oxygenation and regenerative state, the selected endpoint, and follow-up duration. The more informative question is therefore not whether a given radiation modality or organ “has a FLASH effect, “ but where the physical-biological parameter boundaries lie for its appearance, attenuation, or disappearance and what effect magnitude and uncertainty accompany normal-tissue sparing within those boundaries. Preclinical evidence is relatively concentrated in the central nervous system, lung, and selected skin models, but even in the CNS much of the positive normal-tissue evidence derives from otherwise healthy rodent whole-brain models, whereas intracranial tumor-bearing models remain less extensively investigated. Gastrointestinal and hematopoietic systems, fractionated irradiation, and late toxicity remain strongly model- and parameter-dependent. The FLASH phenotype therefore cannot yet be extrapolated directly across organs, platforms, or treatment schedules.

The principal limitation of the evidence base is not simply the number of studies but its composition and limited cross-study comparability. Most biological data still derive from single-center small-animal experiments, with differences in spatial dose distribution, irradiated volume, total dose, temporal structure, anesthesia and oxygenation, biological endpoints, and follow-up that constrain direct comparison of positive and negative findings. Small-animal tumor models may also differ from human tumors in genetic heterogeneity, cell kinetics, fractionation sensitivity, and microenvironmental features including hypoxia, further limiting direct translational extrapolation. Inter-institutional replication, large-animal studies, and human studies with concurrent CDR comparators remain limited. Because this Review uses a problem-oriented narrative framework, our synthesis of the relative strength of organ-specific and mechanistic evidence should not be interpreted as a formal evidence hierarchy or quantitative effect estimate. Most importantly, existing human studies primarily establish clinical feasibility and preliminary safety of UHDR delivery on selected electron and proton platforms; they have not demonstrated reproducible comparative normal-tissue sparing relative to CDR irradiation. Clinical feasibility and short-term safety are necessary prerequisites for further study but cannot substitute for direct evidence of therapeutic gain. Similarly, acute normal-tissue sparing cannot substitute for independent assessment of late toxicity and long-term organ function.

Future research should move beyond threshold seeking and significance-driven reporting toward parameterized, quantitative, and reproducible exposure-effect frameworks. At the physical level, total dose, DPP, intra-pulse dose rate, pulse width and interval, total delivery time, and local voxel dose-accumulation history should be reported consistently and completely, with traceable reference dosimetry, time-resolved beam monitoring, and log-file reconstruction used to define actual physical exposure and its uncertainty. Biological studies should prioritize complete organ-specific dose-response relationships, report effect sizes, confidence intervals, and negative results, and complement isolated histological or short-term surrogate endpoints with organ function, tissue homeostasis, and long-term outcomes. Inter-institutional replication, large-animal validation, and studies using clinically relevant irradiated volumes and fractionation are particularly important for establishing external reproducibility. Mechanistic research should likewise progress from molecular and pathway association toward causal testing: temporal structure should be varied systematically under comparable total dose and spatial dose conditions; oxygen and radical kinetics should be measured in a time-resolved manner where possible; and genetic, pharmacological, and, when appropriate, rescue experiments should test candidate nodes in mitochondrial homeostasis, redox regulation, ferroptosis, immune inflammation, and tissue remodeling while normal-tissue and tumor responses are evaluated in parallel. Only mechanistic effects that are directionally consistent and reproducible across platforms, organs, and experimental systems are likely to support reliable parameter extrapolation or clinical stratification.

From a translational perspective, FLASH-RT should not currently be developed on the premise of broadly replacing conventional radiotherapy. Priority should instead be given to settings in which normal-tissue toxicity is dose limiting, the intended temporal structure can be delivered stably and traceably under clinically relevant anatomy and irradiated-volume conditions, and efficacy and toxicity endpoints are well defined. Indication expansion is best governed by prespecified stage-gated continue/stop criteria: first, establish patient-level accuracy, reproducibility, and auditability of absorbed dose and complete temporal structure; next, confirm reproducible organ-specific normal-tissue sparing under clinically relevant total dose, volume, and fractionation; concurrently demonstrate tumor control that is not inferior to the relevant CDR treatment and exclude unacceptable late toxicity over an organ-appropriate follow-up interval; and finally demonstrate a patient-meaningful comparative therapeutic benefit in an appropriately controlled clinical study. Only after this physical, dosimetric, biological, oncological, and long-term safety evidence chain has been built should irradiated volumes be enlarged, exposure of organs at risk increased, more complex fractionation introduced, or curative-intent studies pursued. The future of FLASH-RT therefore depends less on achieving ever higher nominal dose rates than on establishing an evidence framework that reliably connects actual physical exposure with organ-specific biological responses and long-term patient outcomes, thereby transforming a condition-dependent experimental phenomenon into a predictable, testable, reproducible, and auditable clinical treatment strategy.
